# Aqueous Modification of Chitosan with Itaconic Acid to Produce Strong Oxygen Barrier Film

**DOI:** 10.1021/acs.biomac.1c00216

**Published:** 2021-04-29

**Authors:** Juho Antti Sirviö, Anu M. Kantola, Sanna Komulainen, Svitlana Filonenko

**Affiliations:** † Fibre and Particle Engineering Research Unit, University of Oulu, P.O. Box 4300, FI-90014 Oulu, Finland; ‡ NMR Research Unit, University of Oulu, P.O. Box 3000, FI-90014 Oulu, Finland; § Max Planck Institute of Colloids and Interfaces, Research Campus Golm, 14424 Potsdam, Germany

## Abstract

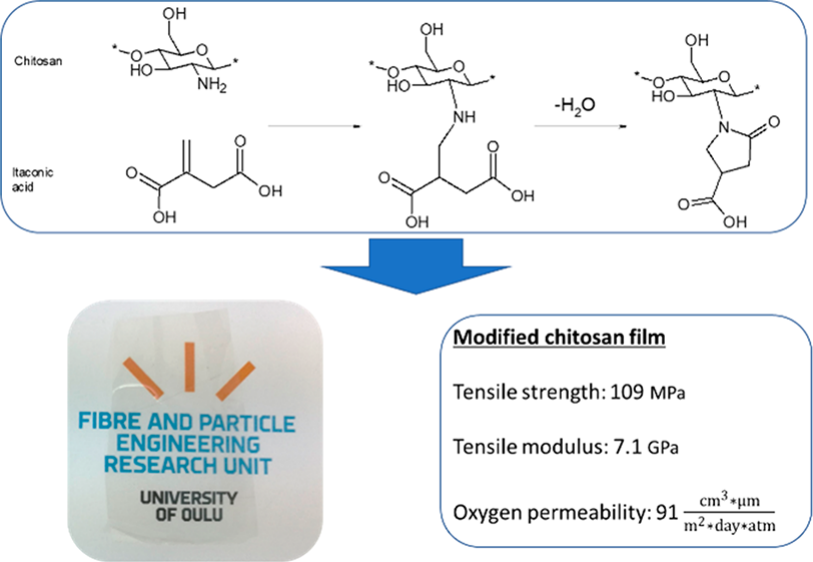

In this study, the chemical modification of chitosan using itaconic acid as a natural-based unsaturated dicarboxylic acid was investigated. In an aqueous environment, the amine group of chitosan reacts with itaconic acid to produce a chitosan derivative with pyrrolidone-4-carboxylic acid group. On the basis of the elemental analysis, 15% of the amine groups of chitosan reacted, thus creating modified chitosan with amine and carboxylic acid functionalities. Due to the presence of amine and carboxylic acid groups, the surface charge properties of the chitosan were notably altered after itaconic acid modification. In an aqueous solution, the modified chitosan exhibited zwitterionic properties, being cationic at low pH and turning anionic when the pH was increased over 6.5, whereas the original chitosan remained cationic until pH 9. Furthermore, it was demostrated that the modified chitosan was suitable for the preparation of a self-standing film with similarly high transparency but notably higher mechanical strength and oxygen barrier properties compared to a film made from the original chitosan. In addition, the thermal stability of the modified chitosan film was higher than that of the original chitosan film, and the modified chitosan exhibited flame-retardant properties.

## 1 Introduction

Natural-based materials and chemicals are currently desired as a replacement for the products derived from nonrenewable fossil-based resources. Replacing high-volume, single-use products, such as packaging as well as materials that easily end up in the environment, including soil stabilizers and water treatment chemicals, is of great interest due to the poor biodegradability and toxicity of fossil-based materials.^1^

Polysaccharides are a large family of sugar-based polymers, and they are widely available in many forms from renewable resources.^2^ Among the most abundant polysaccharides are cellulose, starch, chitin, and chitosan. Cellulose and starch are glucose-based polymers available in plants as structural^3^ and energy storage^4^ components, respectively. Chitin, a polymer of *N*-acetylglucosamine, is the primary component of the cell walls of fungi and can be found in many insects and sea animals.^5^ Chitin can be used to produce chitosan, a unique polysaccharide with amino groups, by alkaline deacetylation. Chitosan can also be found in certain fungi.^6^

Chemical modification of polysaccharides is frequently used to update their properties to meet the demand of the target application.^7^ Most of the polysaccharides contain an abundance of hydroxyl groups, which can be esterified, etherified, and oxidized. The amino groups of chitosan create an exceptional opportunity for chemical modification, and they have been converted, for example, into quaternary amine by methylation,^8^ imidazole by Debus–Radziszewski imidazole synthesis,^9^ and 5-methylpyrrolidinone by reductive amination.^10^

Despite the great advances in the field of chemical modification of polysaccharides, many of the methods are based on the use of hazardous and fossil-based chemicals. The chemicals typically used in polysaccharide modification include halogen-based oxidizers,^11^ acyl^12^ and alkyl^13^ halogens, and oil-based anhydride.^14^ Therefore, there is a need for methods that utilize less hazardous, natural-derived chemicals. Furthermore, performing reactions in sustainable solvents, such as water, is highly desirable.

Itaconic acid is an unsaturated dicarboxylic acid produced in large scale by fermentation of sugars and industrial wastes such as glycerol,^15^ thus being an environmentally friendly building block for polymer synthesis^16,17^ and modification of natural polymers.^18,19^ Due to its unique chemical structure, itaconic acid can react with primary amines by the aza-Michael reaction resulting in the formation of cyclic pyrrolidone-4-carboxylic acid.^20^ The aza-Michael reaction of itaconic acid has previously been utilized in the production of monomers for polyesters^17^ and polyamides.^21^ The reaction between itaconic acid and amines can be conducted in water, further elevating the environmental feasibility of this reaction. To the best of our knowledge, previous investigation on the modification of chitosan with itaconic acid is mainly based on the ionic interaction between two components^22^ and amidation of the carboxylic group of polyitaconic acid and the amine group of chitosan.^23^ In this study, chemical modification of chitosan was studied using itaconic acid in water to produce chitosan with pyrrolidone-4-carboxylic acid functionality. The charge and the film-forming properties of the modified chitosan were investigated and compared to those of the original chitosan.

## 2 Materials and Methods

### 2.1 Materials

Chitosan with a medium molecular weight (190 000–310 000 g/mol) was obtained from Sigma-Aldrich Co. (Germany), itaconic acid was obtained from TCI (Japan), ethanol was obtained from VWR (France), and 0.5 M NaOH was obtained from FF-Chemicals (Finland).

Polydiallyldimethylammonium chloride (polyDADMAC) and polyethylene sodium sulfonate (PES-Na) were obtained from BTG Mütek GmbH (Germany) and were used as polyelectrolyte titrants. For all steps requiring water, unless stated otherwise, deionized water was used.

### 2.2 Modification of Chitosan with Itaconic Acid

Modification of chitosan with itaconic acid was carried out using a method similar to that previously published for the aza-Michael reaction between monomeric amine (ethanolamine) and itaconic acid.^17^ Chitosan (1 g) was mixed with water (100 mL) in a Schott Duran bottle, followed by the addition of 6 g of itaconic acid. The bottle was then shut with a cap and placed in an oil bath at 90 °C, and the reaction mixture was allowed to react under mixing for 24 h. Then, the bottle was removed from the oil bath and the reaction mixture was allowed to cool to room temperature. The pH of the reaction mixture was then adjusted to 8 with the use of 0.5 M NaOH solution. Then, 200 mL of ethanol was added, and the precipitated gel-like product was filtrated and washed with 1 L of ethanol–water mixture (1:1). The product was collected and dried in an oven at 60 °C.

### 2.3 Elemental Analysis

The nitrogen content of the original and modified chitosan was analyzed with a PerkinElmer CHNS/O 2400 Series II elemental analyzer. The degree of substitution (DS) was calculated with eq 1:^24^
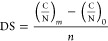
1where (C/N)_*m*_ is the carbon–nitrogen ratio of the chitosan derivative, (C/N)_0_ is the carbon–nitrogen ratio of chitosan, and *n* is the number of carbons introduced during the chitosan derivatization.

### 2.4 Dissolution of Modified Chitosan in Water

Modified chitosan was mixed with water at a consistency of 1% for 24 h at room temperature. The obtained gel was then passed once at a pressure of 1000 bar through the 400 and 200 μm chambers of a microfluidizer (Microfluidics M-110EH-30, USA) to obtain a clear solution. Due to the dilution of the sample during the microfluidization, the consistency of the modified chitosan solution was determined by drying a known amount of solution at 100 °C in oven for 24 h.

### 2.5 Transmission Electron Microscope

A transmission electron microscope (TEM, JEOL JEM-2200FS, Japan) was used to investigate the possible presence of nanometric size particles in an aqueous solution of the modified chitosan after microfluidization. The sample was prepared by adding 7 μL of a diluted poly-l-lysine solution, 7 μL of a diluted modified chitosan suspension, and 7 μL of 2% uranyl acetate on a carbon-coated copper grid. Between adding the chemicals, each of these solutions was allowed to remain on the grid for approximately 30 s, and after that, they were dried with a filter paper. Additional sample was prepared in a similar manner but without poly-l-lysine.

### 2.6 Optical Transmittance of Solutions

Ultraviolet–visible (UV–vis) spectroscopy was used to investigate the transparency of original and modified chitosan solutions. The original chitosan (1%) was dissolved in 2% acetic acid solution and further diluted to 0.1% with water, and the modified chitosan was also diluted to 0.1% with water. UV–vis spectra (190–800 nm) were recorded with a UV–visible spectrometer (Shimadzu, Japan) using quartz cuvettes.

### 2.7 Diffuse Reflectance Infrared Fourier Transform Spectroscopy

The chemical characterization of the original chitosan and the modified chitosan was performed with diffuse reflectance infrared Fourier transform (DRIFT) spectroscopy. The spectra were collected with a Bruker Vertex 80v spectrometer (USA) from dried samples. The spectra were obtained in the 600–4000 cm^–1^ range, and 40 scans were taken at a resolution of 2 cm^–1^ from each sample.

### 2.8 Nuclear Magnetic Resonance Spectroscopy

Solid-state ^13^C CP-MAS (cross-polarization magic angle spinning) NMR spectra were measured both from the original chitosan and from the freeze-dried modified chitosan with a Bruker Avance III 300 spectrometer having a ^13^C resonance frequency of 75.5 MHz. The samples were packed into 7 mm zirconia rotors, and the spinning frequency was 5 kHz. Tetramethylsilane at 0 ppm was used as an external standard. Spectra were acquired with 16 384 scans, 4 s repetition rate, 1.5 ms variable amplitude contact pulse, and SPINAL-64 decoupling during the acquisition.

### 2.9 Zeta Potential

Zeta potential was measured from the original chitosan dissolved in 2% acetic acid and the modified chitosan after dissolution in water. Initial values of the pH of the original chitosan solution (3.71) and the modified chitosan (8.12) were determined before measurements with a one-electrode pH meter (WTW MultiLine P4, Xylem Analytics Germany). The pH of both solutions was adjusted to 3 with 0.01 or 0.1 N HCl correspondingly to avoid excessive dilution of the samples. Further titration was carried out under constant stirring with the use of 0.1 or 0.01 N NaOH in different pH regions in order to achieve minimal volume change due to the addition of titrant. The zeta potential values at specific pH were measured with a Zetasizer Nano ZS, Malvern Instruments (Malvern Panalytical, U.K.), and the results are presented as the average of three measurement.

### 2.10 Charge Density

The surface charge densities of the original chitosan and the modified chitosan were determined by using the polyelectrolyte titration method through a particle charge detector (BTG Mütek PCD-03, Germany). A 10 mL volume of modified chitosan suspension (at 0.01 wt %) in a buffer solution was titrated with polyDADMAC or PES-Na (1 mequiv/L). The charge density was calculated on the basis of titrant consumption, and results are presented as the average of two measurements.

### 2.11 Fabrication of Self-Standing Films

Self-standing films were produced from the original chitosan dissolved in 2% acetic acid (1% chitosan) and the modified chitosan after dissolution in water by the solvent-casting method. Both samples were cast on polystyrene trays, and solvent was allowed to evaporate in a fume hood at room temperature. Films were then peeled from trays and transferred to an air-conditioned room (relative humidity of 50% and temperature of 23 °C) for at least 48 h before measurements.

### 2.12 Optical Transmittances of Films

The transmittances of the original and modified chitosan films were measured in the wavelength range 200–800 nm with a UV–vis spectrometer (Shimadzu, Japan). In order to ensure that the films were perpendicularly aligned against the incoming beam and to avoid wrinkling, the films were put between two quartz glass slides before they were set up in a cuvette stand.

### 2.13 Tensile Test

The tensile properties of the films were measured with a universal testing device (Instron 5544, USA) at controlled environmental conditions (relative humidity of 50% and temperature of 23 °C). Prior to measurement, films were cut into strips at a length of 70 mm and a width of 5 mm, and the average thickness of each strip was measured at three random positions with a thickness gauge (FT3 Precision Thickness Gauge, Hanatek Instruments, U.K.). Five samples of each film were measured with a 2 kN force sensor, at a gauge length of 40 mm and a strain speed of 5 mm min^–1^ until a break occurred.

### 2.14 Oxygen Barrier Measurement

The oxygen transmission rate (OTR) values of the films were measured with a MOCON OX-TRAN 2/20 (Minneapolis, MN), and the oxygen permeability (OP) was calculated by multiplying the OTR values with the thickness of the film and dividing it by the difference in the partial pressure of the oxygen gas between the two sides of the film. Prior to the measurement, samples were glued between two aluminum plates having a measurement area of 5 cm^2^. During measurement, the films were exposed to 100% oxygen on one side and to oxygen-free nitrogen gas (98% nitrogen and 2% hydrogen) on the other side. Three samples of each film were measured at 23 °C, with a relative humidity of 50% and at normal atmospheric pressure.

### 2.15 Thermal Properties

Thermal properties of the original and modified chitosan films were characterized with a NETZSCH STA 449 F3 (Germany) thermal analyzer. Approximately 5 mg of the sample was heated in an aluminum oxide pan from 30 to 900 °C at a heating rate of 10 °C/min under the flow of dynamic air (flow rate of 60 mL/min).

### 2.16 Statistical Analysis

One-way analysis of variance (ANOVA) was conducted by using OriginPro 2020 to determine the statistical significance (*p* < 0.05) of the tensile and barrier test results.

## 3 Results and Discussion

### 3.1 Itaconic Acid Modification of Chitosan

**Scheme 1:**
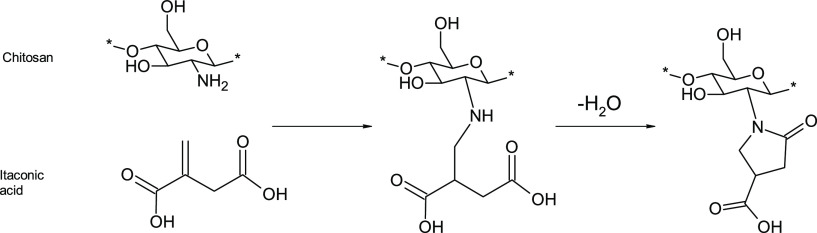
Chemical Modification of Chitosan with Itaconic Acid Acetylated groups of chitosan are not shown for the sake of clarity.

The chemical modification of chitosan was attempted in an aqueous itaconic acid solution. Due to the protonation of amine groups, chitosan dissolved in an itaconic acid solution during the reaction. After 24 h of reaction time at 90 °C, no precipitation of chitosan was observed after adjustment of the pH to neutral, contrary to chitosan dissolved in acetic acid (i.e., neutralization of chitosan–acetic acid solution leads to precipitation of chitosan). The solution became slightly turbid around pH 6; however, after further increase in pH, the solution changed to clear again. The addition of ethanol resulted in precipitation of a gel-like product, which was purified by filtration and washing with aqueous ethanol. Based on the elemental analysis, the DS of the product was 0.1. As the degree of deacetylation of the original chitosan was 68%, around 15% of amine groups reacted with itaconic acid. The low conversion of amine groups to the corresponding pyrrolidone-4-carboxylic acid is most likely due to the protonation of amine in acidic medium, inhibiting the aza-Michael reaction. However, carboxylic acid and amines are weak acid and base, respectively, and they exist in water in equilibrium between salt and free acid and base. The presence of free amine groups allows the chemical modification of chitosan to take place. The proposed reaction and the product are presented in Scheme 1.

The dried product swelled significantly in water; however, no complete dissolution was observed after mixing for 24 h at room temperature, and the product remained in a clumpy, gel-like state. After the gel was passed once through the microfluidizer, a clear mixture was obtained. The transparency of the modified chitosan solution after fluidization was over 90% in the visible light region, similar to the solution of the original chitosan in an aqueous acetic acid (Figure S1). No particles were observed in TEM imaging, indicating that microfluidization treatment helped to dissolve modified chitosan, instead of disintegrating it into nanosized chitosan particles. Poor dissolution of the modified chitosan in water after drying might be due to the formation of ionic cross-linking between anionic carboxylate and cationic amine groups as well as hydrogen bonding within modified chitosan molecules. Nevertheless, the mechanical force applied by microfluidization is strong enough to enable dissolution of the modified chitosan in water.

### 3.2 Chemical Analysis of the Modified Chitosan

**Figure 1:**
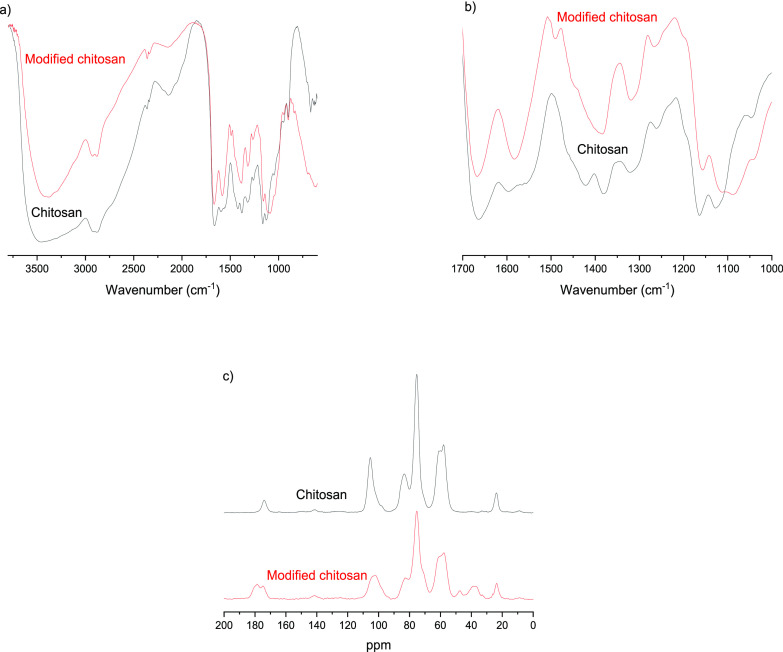
(a) DRIFT spectra, (b) fingerprint region of the DRIFT spectra, and (c) NMR spectra of original chitosan and modified chitosan.

The chemical modification of chitosan with itaconic acid was characterized using DRIFT spectroscopy. The spectrum of the original chitosan showed characteristic peaks of chitosan: bands around 3500 cm^–1^ (OH and NH stretching), 1664 cm^–1^ (C=O vibration of amide), and 1595 cm^–1^ (NH_2_ deformation) (Figure 1a).^25^ The most notable change in the chitosan spectrum after itaconic acid modification can been seen as the appearance of strong peaks at 1583 and 1384 cm^–1^ (Figure 1b). New peaks are associated with the antisymmetric and symmetric stretching of the carboxylate group. The small peak at 1489 cm^–1^ originates from CH_2_ in the pyrrolidone ring.^26^ Changes in the DRIFT spectrum strongly indicate that chitosan is chemically modified by itaconic acid. Due to the use of water as a solvent, the esterification of chitosan with itaconic acid was assumed not to take place, and it was confirmed by the absence of an ester bond around 1720 cm^–1^ in DRIFT spectra.

Solid-state NMR gave further indications of the chemical modification of chitosan by itaconic acid. The C=O peak of the original chitosan appeared at 174 ppm, and after itaconic acid modification, two peaks can be observed in this region (at 175 and 179 ppm) (Figure 1c). These peaks can be attributed to C=O peaks of formed carboxylate and cyclic amide of pyrrolidone-4-carboxylic acid, as well as the original C=O amide peak in chitosan. The new peaks between around 38 and 47 ppm are related to the CH_2_ and CH groups of the pyrrolidone ring.^17^ Broadening of the anomeric proton peak of chitosan at 105 ppm indicated that an additional C1 carbon species is presented in the modified chitosan compared to the original chitosan. Furthermore, the absence of peaks related to alkene carbon around 130 ppm in the spectrum of modified chitosan indicated that no significant amount of nonreacted itaconic acid existed in the product after washing.

### 3.3 Charge Properties of the Modified Chitosan

**Figure 2:**
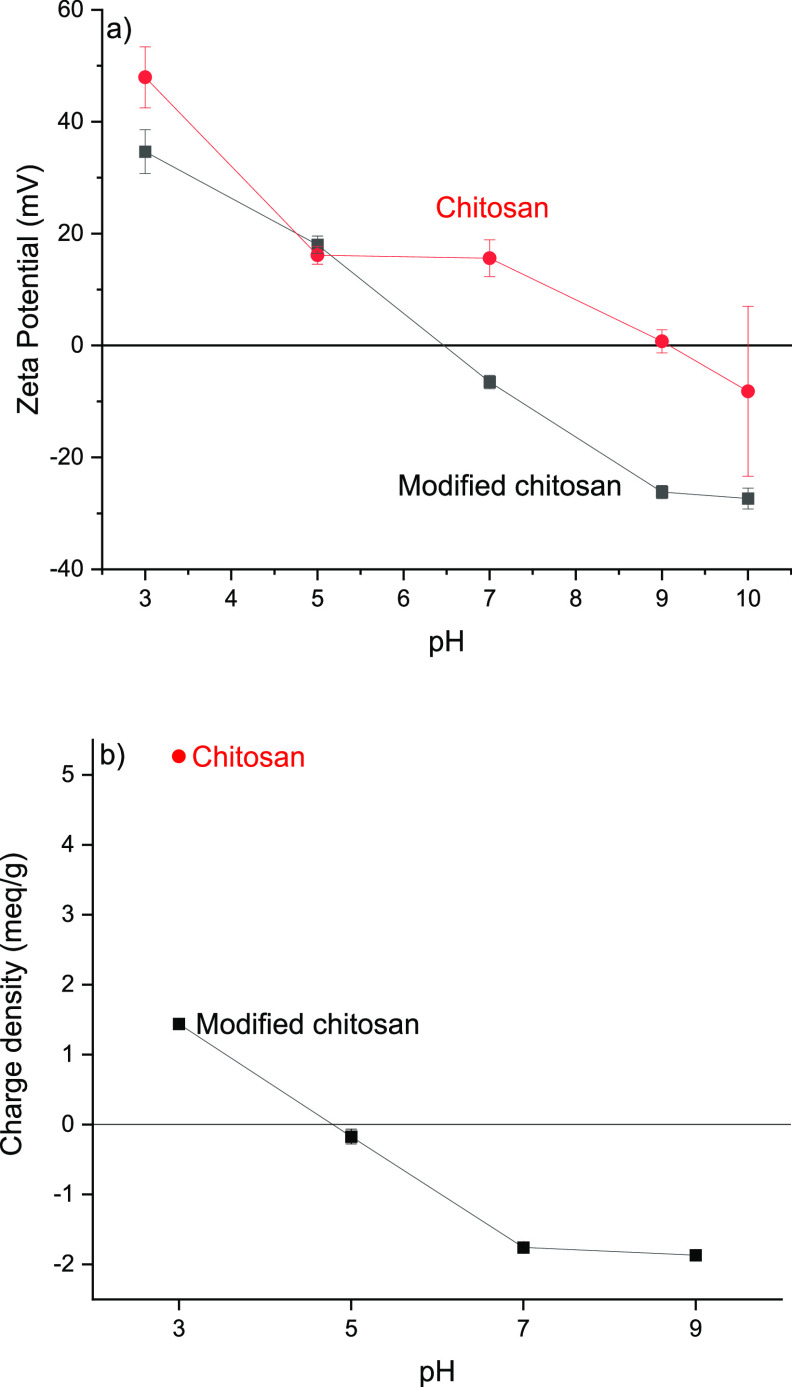
(a) Zeta potentials and (b) charge densities of original and modified chitosans as a function of pH.

The changes in the surface chemistry of the chitosan during the itaconic acid modification were characterized with zeta potential measurement. Due to the amine groups, unmodified chitosan exhibits cationic surface charge at acidic pH. The zeta potential value of chitosan was 48 mV at pH 3 and dropped to 16 mV for pH 5 and 7 (Figure 2a). The isoelectric point, that is, the point where the surface charge was zero, was found to be around pH 9 for the original chitosan.

Similar to the unmodified chitosan, the zeta potential of itaconic acid modified chitosan was positive at pH 3, lower compared to the original chitosan (35 vs 48 mV) (Figure 2a). The zeta potential value of itaconic acid modified chitosan gradually decreased when the pH increased, and a zeta potential of −6 mV was observed at neutral condition. The further decrease of the pH resulted in the drop of zeta potential value to around −26 mV at pH 9. When the pH was increased to 10, no significant change was observed in zeta potential value compared to pH 9. The isoelectric point of the modified chitosan was found to be around 6.5, being notably lower than the unmodified chitosan.

The amine groups of chitosan have been reported to have p*K*_a_ values of around 6.5^27,28^ indicating that most of the amine groups of original chitosan are protonated below pH 6 and the isoelectric point of should be close to this pH. Previously, the isoelectric point of chitosan in zeta potential measurement has been observed around pH 6.^29^ However, zeta potential measurement is sensitive to the structure and conformation of the polymer, and different isoelectric points of chitosan have been reported. Chitosan with a deacetylation degree of 28.8% showed a zero zeta potential value between pH 7.5 and 8, whereas chitosan with a deacetylation degree of 15% exhibited an isoelectric point at higher pH (around 8).^30^ Furthermore, ionic strength has been shown to slightly modify the isoelectric point of chitosan. When measured in 0.01 M NaCl solution, chitosan from prawn shell was reported to have an isoelectric point at pH 8.2 and at 0.001 M it was at pH 8.5,^31^ results that are close to the isoelectric point observed in the current study.

The polyelectrolyte titration further suggested the changes of the surface charge of modified chitosan from positive to negative as a function of pH (Figure 2a). At low pH, the charge of the modified chitosan was positive (1.48 mequiv/g at pH 3), whereas the charge of the original chitosan was 5.27 mequiv/g at the same pH. The charge density of the modified chitosan gradually decreased when the pH increased from 3 to 7 and remained consistent from pH 7 to 9 (around −1.7 mequiv/g).

Although the isoelectric point and the pH where the charge of the modified chitosan changed from positive to negative value in polyelectrolyte titration were slightly different, most likely due to the differences in measurement, they both strongly indicated that the original chitosan and the modified chitosan have different charge properties in water solution as a function of pH. The original chitosan has an abundance of primary amine groups in its structure (4.3 mmol/g determined by conductometric titration), which are protonated at low pH, thus creating positive surface charge. The surface charge decreases when the pH increases due to the deprotonation of amine groups.

On the other hand, the chemical modification of chitosan by itaconic acid resulted in the formation of one carboxylic acid group from one amine group. Since only part of the amine groups took part in the reaction, the surface charge of the modified chitosan was still positive at low pH due to the protonation of both carboxylic acid and amines. When the pH increases, the amine groups are deprotonated, similar to the original chitosan, thus decreasing the positive charge. At the same time, carboxylic acid is also deprotonated, creating negative charge on the surface. At the isoelectric point, the sum of the protonated amines and deprotonated carboxylic acids is equal, thus resulting in zero net surface charge. When the pH is further increased, there exist more deprotonated carboxylic acids compared to protonated amines, and the surface of the modified chitosan becomes negatively charged.

Chitosan with positive and negative chemical groups (zwitterionic) has previously been obtained using carboxymethylation^32^ and (2,2,6,6-tetramethylpiperidin-1-yl)oxyl (TEMPO)-mediated oxidation,^33^ with both utilizing halogen-based, hazardous stoichiometric chemicals. The succinylation of chitosan with succinic anhydride is a sustainable alternative for carboxymethylation and TEMPO-mediated oxidation, but it involves the use of organic solvents (e.g., dimethyl sulfoxide^34^ and dimethylformamide^35,36^). Therefore, itaconic acid modification can be seen as a sustainable method for the introduction of carboxylic acid moiety in chitosan. Zwitterionic chitosan has previously been investigated, for example, to prepare emulsion stabilizers^37,38^ and in medical applications.^39,40^ Due to the zwitterionic characteristic, it can be assumed that modified chitosan could be used in metal adsorption,^41^ for example, in water purification and medical applications.^42^

### 3.4 Preparation of a Film from Modified Chitosan

**Figure 3:**
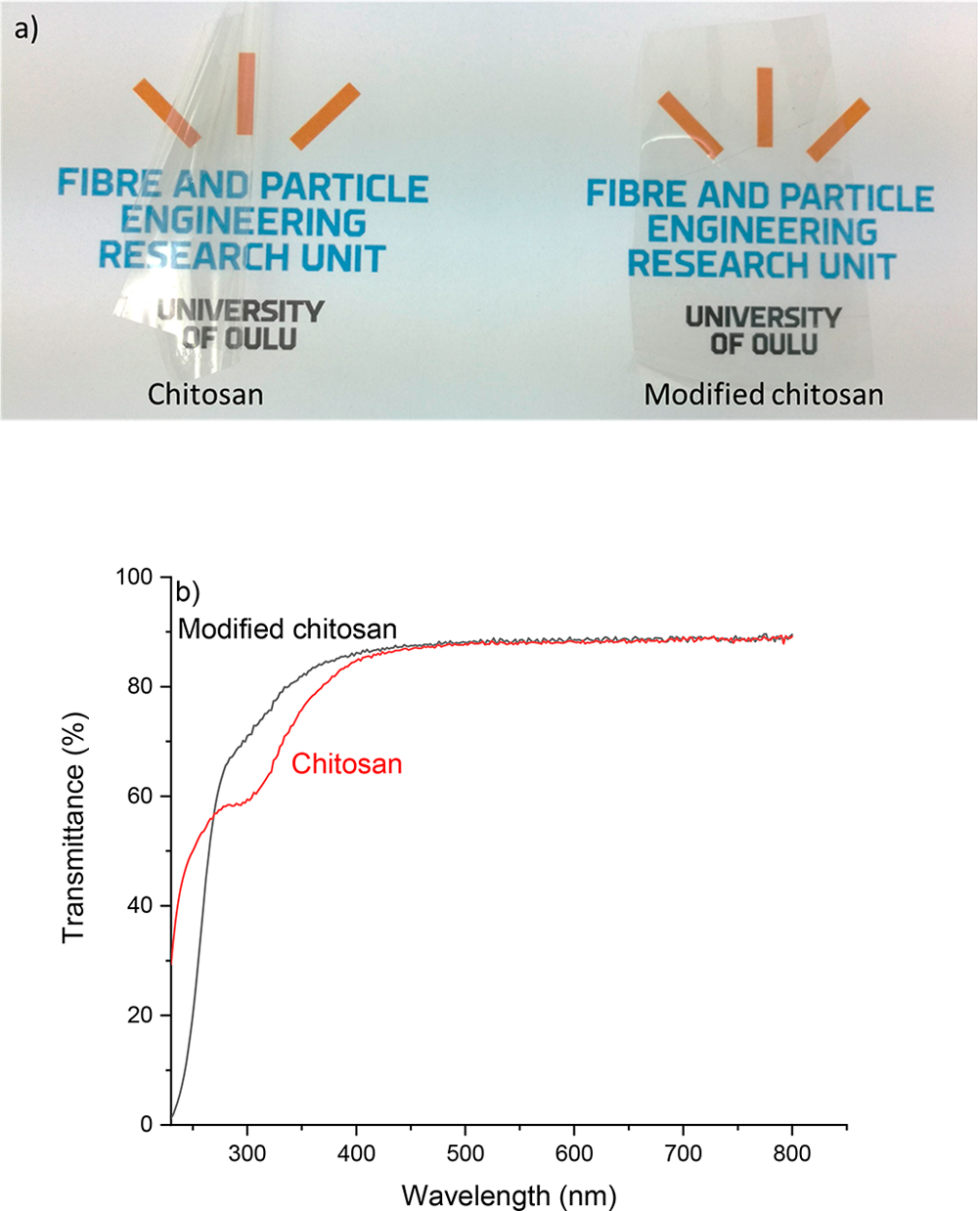
(a) Photograph and (b) UV–vis spectra of original (A) and modified chitosan (B) films demonstrating the high transparency of both films and curving of the original chitosan film.

Self-standing films were produced from the original chitosan and the modified chitosan by solvent casting. Both films appeared similarly transparent; however, the chitosan film was prone to curving at a relative humidity of 50% after removal from polystyrene tray, whereas the modified chitosan stayed flat (Figure 3). The curving of the chitosan film indicates that it has lower dimension stability compared to the modified chitosan. The different dimension stabilities between the two samples might originate from differences in their water absorption and swelling properties,^43^ previously observed in cellulosic nanomaterials.^44^

### 3.5 Tensile Properties of Modified Chitosan Film

Chitosan is well-known to produce self-standing films with good mechanical properties, and the tensile strength generally ranges from 10 to 90 MPa, depending on the film-forming solution and properties of chitosan.^45^ Here, the tensile strength of the film produced from the original chitosan was 53 MPa (Table 1), being well in line with results reported in the literature. Surprisingly, the mechanical properties of the modified chitosan films were found to be significantly higher than those of the original chitosan films. The tensile strength and modulus of the modified film were 109 MPa and 7.1 GPa (Table 1), both being around 2 times higher than those of the film obtained from the original chitosan. Both films exhibited similar elongation, and there was no statistical significance between strain values. In addition, the densities of original chitosan and modified chitosan films were close to each other, and differences in the specific tensile properties were in a similar range compared to tensile properties. For example, the tensile strength of modified chitosan was around 2.1 times higher compared to that of original chitosan, whereas the specific tensile strength of modified chitosan film was 1.8 times higher than that of chitosan film.

**Table 1:** Density and Mechanical and Oxygen Barrier Properties of Original and Modified Chitosan Films

		mechanical properties							
	density (g/m^3^)	tensile strength (MPa)	specific tensile strength (kN·m/kg)	tensile modulus (GPa)	specific tensile modulus (MN·m/kg)	strain (%)	yield strength (MPa)	specific yield strength (kN·m/kg)	oxygen barrier, OP (cm^3^·μm/m^2^·day·atm)
chitosan	0.97	109 ± 14^a^	113 ± 15^a^	7.1 ± 1.2^a^	7.3 ± 1.2^a^	4.2 ± 1.9^a^	97 ± 14^a^	100 ± 14^a^	91 ± 19^a^
modified chitosan	0.83	53 ± 17^b^	64 ± 17^b^	3.7 ± 0.8^b^	4.7 ± 1.0^b^	5.5 ± 1.8^a^	52 ± 13^b^	55 ± 13^b^	191 ± 39^b^

Different superscript letters within the same column are significantly different at 0.05 level based on the one-way ANOVA

The stress–strain curves of original chitosan and modified chitosan films indicated that they have slightly different behaviors related to strain-induced stress. After the yield point, there exists a drop in the stress–strain curve of the original chitosan film until the lower yield point is reached (Figure S2a). The upper yield point indicates the maximum force that can be applied before chitosan chains start to slip among each other, causing the permanent deformation of the film. The drop in the stress–strain curve is the result of the loss of the resistance of the film toward the external force caused by the slippage of the polymer chains.^46^ After the lower yield point, strain-hardening regions are observed. The strain hardening is due to the reorientation of the polymer, for example, straightening of the curved chains, resulting in the formation of new bonding between polymers.^47^

The stress–strain curve of the modified chitosan shows the direct transition from the yield point to the strain-hardening region (Figure S2b). The reason for the absence of a lower yield point is unknown; however, it might be that, due to the presence of both anionic and cationic groups, there exists dynamic ionic cross-linking^48^ between the modified chitosan molecules and the rearrangement of the ionic cross-linking is a fast process and, therefore, there exists no drop in the stress–strain curve of the modified chitosan. However, since the pH of the modified chitosan solution was only adjusted after modification, more thorough studies should be conducted to investigate the effect of ionic cross-linking on the mechanical properties of the modified chitosan film.

The direct transition from yield point to strain-hardening region is very common for cellulose nanofiber (CNF) films. In the case of CNF films, the strain hardening is a prominent effect, and the ultimate tensile strength of CNF films can be over 2 times higher compared to yield strength.^49,50^ For example, holo-CNFs have been used to produce one of the strongest CNF films, having a tensile strength of 320 MPa,^51^ a value that is almost 3 times higher compared to that of the film produced from the modified chitosan. Nevertheless, the yield strength of holo-CNF film was 120 MPa, thus being only moderately higher compared to that of the modified chitosan film (97 MPa). Furthermore, when taking into account the thickness of the films, the specific tensile yield of the modified chitosan film was higher than that of holo-CNF films (100 kN·m/kg for modified chitosan vs 81 kN·m/kg for holo-CNF).

As stated above, after the yield point, a material undergoes permanent deformation, leading to the changes in its structure. The permanent deformation can be adverse in many applications, such as electronics, as it could lead to dysfunction and replacement of the component. Therefore, together with very high transparency, the modified chitosan film could be a similar or even better candidate than CNF films as a supporting layer for electronics, such as flexible solar cell^52,53^ and organic light-emitting diodes.^54^ As stated above, modified chitosan has a yield strength that is in line with that of high-strength CNFs, whereas the visible light transparency of modified chitosan is very high, which in the case of CNFs is generally obtained using hazardous halogenated chemicals.^55^ However, before the modified chitosan could be used in electronics, a problem arising from high hygroscopy and poor water tolerance (CNF films exhibit a similar problem^56^) should be solved. The water tolerance of modified chitosan films could be improved by laminating^57^ with hydrophobic materials or using heat-induced covalent cross-linking by amination of amine and carboxylic groups.^58,59^

### 3.6 Oxygen Barrier Properties of the Modified Chitosan Film

The oxygen barrier properties of films are important in many applications as many foods and electronic components are prone to oxygen-induced damage. Chitosan has been reported to have a low OP; thus, it has good oxygen barrier properties. The film from the original chitosan exhibited an OP value of 191 cm^3^·μm/m^2^·day·atm and can be described as high oxygen barrier (OP values between 40 and 400 cm^3^·μm/m^2^·day·atm^60^) (Table 1). However, the modified chitosan showed an over 2 times lower OP value, indicating that, in addition to being a significantly stronger material, the modified chitosan film is a better barrier against oxygen than the original chitosan.

The oxygen barrier properties of original and modified chitosan films were significantly better than those of common hydrophobic plastics used in everyday packaging, including poly(ethylene terephthalate), which has an OP value in the range 1000–5000 cm^3^·μm/m^2^·day·atm.^60^ Furthermore, chitosan-based films had OP values similar to that of polyvinylidene chloride (10–300 cm^3^·μm/m^2^·day·atm^61^), which is used as an oxygen barrier material. Nevertheless, excellent fossil-based oxygen barriers, such as ethylene vinyl alcohol^60^ and poly(vinyl alcohol),^62^ have notable lower OP values compared to modified chitosan films.

Compared to other bio-based oxygen barriers, original and modified chitosan films exhibit superior barrier properties compared to amylopectin and amylose^63^ and lower OP values compared to cellophane.^60^ Cellulosic nanomaterials (CNFs and cellulose nanocrystals (CNCs)) are described as high oxygen barrier materials due to their high polarities and degrees of crystallinity. Nevertheless, the oxygen barrier properties of films made from cellulosic nanomaterials depend on the different nanomaterial and film preparation methods. For example, in the case of TEMPO-oxidized CNF, the use of sodium salt as counterion resulted in a film with a higher OP value (250 cm^3^·μm/m^2^·day·atm) compared to chitosan films produced here. However, the change of counterion to calcium greatly enhanced the oxygen barrier properties of a CNF film, with an OP value of 3.6 cm^3^·μm/m^2^·day·atm.^64^ Higher OP values compared to those for modified chitosan films are reported for CNF films produced by the homogenization of cellulose pulp (357–510 cm^3^·μm/m^2^·day·atm)^65^ and films made from *tert*-butylamino-CNCs (250 cm^3^·μm/m^2^·day·atm).^66^ On the other hand, several types of CNF films have been reported to have better oxygen barrier properties compared to modified chitosan films (OP values below 50 cm^3^·μm/m^2^·day·atm).^55,67−69^

### 3.7 Thermal Properties of the Modified Chitosan Film

**Figure 4:**
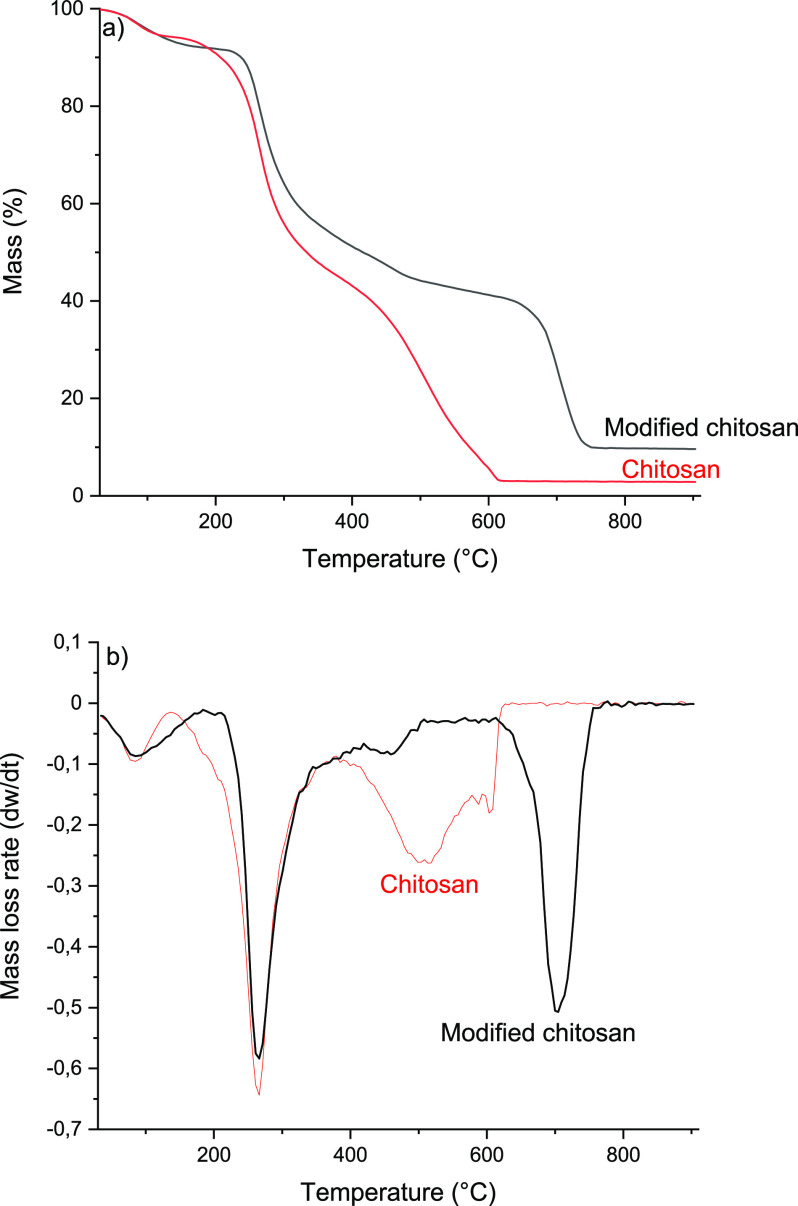
(a) Thermogravimetric analysis and (b) first-derivative curves of original chitosan and modified chitosan.

In addition to the higher strength and better oxygen barrier properties, the modified chitosan exhibited improved thermal stability compared to the original chitosan film (Figure 4). Both films showed the first region of weight loss almost immediately after the start of heating until around 150 °C. The initial weight loss is attributed to the removal of absorbed water from the hydrophilic polysaccharide structure, and no degradation of polymer structure is assumed to take place at this point.^70^ The onset temperature of the degradation of the original chitosan film was at 224 °C, and the maximum thermal degradation rate was observed at 266 °C. During the first degradation step, the dehydration of the saccharide ring, depolymerization, and decomposition of acetylated and deacetylated unit begin, resulting in the formation of volatile components.^71^ The second degradation step begins around 456 °C, and the maximum degradation rate is at 500 °C. The second degradation step is related to the thermal oxidation of the remaining material, and chitosan is burned without notable residue.

Several chemical modifications have been shown to decrease the thermal stability of chitosan.^72,73^ However, after itaconic acid modification, the onset temperature of the chitosan increased over 20 °C to 243 °C compared to the original chitosan film. However, the maximum degradation rate of the modified chitosan was similar to that of the original chitosan. A larger change can be observed in the temperature related to the thermooxidation process, as in the case of the modified chitosan. The second degradation begins at 673 °C, and the maximum degradation rate is at 700 °C, being around 200 °C higher temperatures compared to those for the original chitosan film. Furthermore, compared to the original chitosan, which was burned almost completely, there was around 11% mass residue (the amount of residual mass was compared to the mass of the sample at 200 °C, corresponding to the mass of the sample without absorbed water) at the end of the measurement in the case of the modified chitosan.

Previously, the introduction of carboxylate group into chitosan by TEMPO-laccase oxidation^74^ or carboxymethylation^75^ with chloroacetic acid has been shown to decrease the thermal stability of chitosan. Both TEMPO-laccase oxidation and chloroacetic acid based carboxymethylation result mainly in O-substitution of chitosan (i.e., substitution occurs on hydroxyl groups of chitosan), although some degree of N-substitution (i.e., substitution of amine group) also takes place in the case of carboxymethylation. On the other hand, the selective carboxymethylation of amine groups of chitosan (i.e., N-substitution) with glyoxylic acid by reductive amination has been shown to increase the thermal stability of chitosan.^76^ Therefore, it can be assumed that the higher thermal stability of the modified chitosan compared to chitosan is due to the selective formation of pyrrolidone-4-carboxylic acid, thus reducing the amount of amine groups.

The shift of the second degradation step (i.e., thermooxidation) to a significantly higher temperature after itaconic acid modification indicates that the modified chitosan film exhibited fire-retardant properties. The introduction of pyrrolidone-4-carboxylic acid could result in acid-catalyzed dehydration of the chitosan backbone during heating and the formation of char.^77^ The large quantity of amine groups results in the formation of volatile, nonflammable gas (ammonia), working as a blowing agent.^78^ Together with char formation and the presence of a blowing agent, a protective charring layer is formed, preventing the ignition of the rest of the material. The formation of char is evident in the presence of a notable residual mass even at 900 °C in the case of modified chitosan.

## 4 Conclusions

Chitosan was successfully modified using natural-based itaconic acid to produce a chitosan derivative with both amine and carboxylic acid functionalities. The dual functionality of the modified chitosan resulted in a zwitterionic behavior in water, and the modified chitosan could thus be used in water treatment applications. Furthermore, the modified chitosan showed excellent film-forming properties, and the film exhibited high mechanical strength and good oxygen barrier properties. Hence, the modified chitosan could also be used in packaging as well as in flexible electronics. However, further studies, such as reaction optimization, should be conducted to fully discover the potential of the modified chitosan in various applications.

## Supporting Information Available

UV–vis spectra and stress–strain curves of original and modified chitosans (PDF)

## References

[ref1] Xiong B.; Loss R. D.; Shields D.; Pawlik T.; Hochreiter R.; Zydney A. L.; Kumar M. Polyacrylamide Degradation and Its Implications in Environmental Systems. Npj Clean Water 2018, 1 (1), 1–9. 10.1038/s41545-018-0016-8.

[ref2] Ulvskov P. Annual Plant Reviews, Plant Polysaccharides: Biosynthesis and Bioengineering; John Wiley & Sons: 2010.

[ref3] Brown R. M. J.; Saxena I. M. Cellulose: Molecular and Structural Biology: Selected Articles on the Synthesis, Structure, and Applications of Cellulose; Springer Science & Business Media: 2007.

[ref4] Zobel H. F. Molecules to Granules: A Comprehensive Starch Review. Starch - Stärke 1988, 40 (2), 44–50. 10.1002/star.19880400203.

[ref5] Tang W. J.; Fernandez J. G.; Sohn J. J.; Amemiya C. T. Chitin Is Endogenously Produced in Vertebrates. Curr. Biol. 2015, 25 (7), 897–900. 10.1016/j.cub.2015.01.058. 25772447 PMC4382437

[ref6] Abo Elsoud M. M.; El Kady E. M. Current Trends in Fungal Biosynthesis of Chitin and Chitosan. Bull. Natl. Res. Cent. 2019, 43 (1), 59 10.1186/s42269-019-0105-y.

[ref7] Cumpstey I. Chemical Modification of Polysaccharides. ISRN Organic Chemistry 2013, 2013, 417672 10.1155/2013/417672. 24151557 PMC3787328

[ref8] Curti E.; de Britto D.; Campana-Filho S. P. Methylation of Chitosan with Iodomethane: Effect of Reaction Conditions on Chemoselectivity and Degree of Substitution. Macromol. Biosci. 2003, 3 (10), 571–576. 10.1002/mabi.200300030.

[ref9] Sirviö J. A.; Visanko M.; Liimatainen H. Synthesis of Imidazolium-Crosslinked Chitosan Aerogel and Its Prospect as a Dye Removing Adsorbent. RSC Adv. 2016, 6 (61), 56544–56548. 10.1039/C6RA08301C.

[ref10] Muzzarelli R. A. A.; Ilari P.; Tomasetti M. Preparation and Characteristic Properties of 5-Methyl Pyrrolidinone Chitosan. Carbohydr. Polym. 1993, 20 (2), 99–105. 10.1016/0144-8617(93)90084-H.

[ref11] Isogai A.; Saito T.; Fukuzumi H. TEMPO-Oxidized Cellulose Nanofibers. Nanoscale 2011, 3 (1), 71–85. 10.1039/C0NR00583E. 20957280

[ref12] Maim C. J.; Mench J. W.; Kendall D. L.; Hiatt G. D. Aliphatic Acid Esters of Cellulose. Preparation by Acid-Chloride-Pyridine Procedure. Ind. Eng. Chem. 1951, 43 (3), 684–688. 10.1021/ie50495a033.

[ref13] Ding F.; Qian X.; Zhang Q.; Wu H.; Liu Y.; Xiao L.; Deng H.; Du Y.; Shi X. Electrochemically Induced Reversible Formation of Carboxymethyl Chitin Hydrogel and Tunable Protein Release. New J. Chem. 2015, 39 (2), 1253–1259. 10.1039/C4NJ01704H.

[ref14] Zuo Y.; Gu J.; Yang L.; Qiao Z.; Tan H.; Zhang Y. Synthesis and Characterization of Maleic Anhydride Esterified Corn Starch by the Dry Method. Int. J. Biol. Macromol. 2013, 62, 241–247. 10.1016/j.ijbiomac.2013.08.032. 23999015

[ref15] Yang J.; Xu H.; Jiang J.; Zhang N.; Xie J.; Wei M.; Zhao J. Production of Itaconic Acid Through Microbiological Fermentation of Inexpensive Materials. J. Bioresour. Bioprod. 2019, 4 (3), 135–142. 10.12162/jbb.v4i3.001.

[ref16] Tian Y.; Wang Q.; Cheng J.; Zhang J. A Fully Biomass Based Monomer from Itaconic Acid and Eugenol to Build Degradable Thermosets via Thiol–Ene Click Chemistry. Green Chem. 2020, 22 (3), 921–932. 10.1039/C9GC03931G.

[ref17] Qi P.; Chen H.-L.; Nguyen H. T. H.; Lin C.-C.; Miller S. A. Synthesis of Biorenewable and Water-Degradable Polylactam Esters from Itaconic Acid. Green Chem. 2016, 18 (15), 4170–4175. 10.1039/C6GC01081D.

[ref18] Sabaa M. W.; Mokhtar S. M. Chemically Induced Graft Copolymerization of Itaconic Acid onto Cellulose Fibers. Polym. Test. 2002, 21 (3), 337–343. 10.1016/S0142-9418(01)00094-0.

[ref19] Soto D.; Urdaneta J.; Pernia K.; León O.; Muñoz-Bonilla A.; Fernández-García M. Itaconic Acid Grafted Starch Hydrogels as Metal Remover: Capacity, Selectivity and Adsorption Kinetics. J. Polym. Environ. 2016, 24 (4), 343–355. 10.1007/s10924-016-0780-9.

[ref20] Paytash P. L.; Sparrow E.; Gathe J. C. The Reaction of Itaconic Acid with Primary Amines. J. Am. Chem. Soc. 1950, 72 (3), 1415–1416. 10.1021/ja01159a520.

[ref21] Ali M. A.; Tateyama S.; Kaneko T. Syntheses of Rigid-Rod but Degradable Biopolyamides from Itaconic Acid with Aromatic Diamines. Polym. Degrad. Stab. 2014, 109, 367–372. 10.1016/j.polymdegradstab.2014.05.031.

[ref22] Milosavljević N. B.; Milašinović N. Z.; Popović I. G.; Filipović J. M.; Kalagasidis Krušić M. T. Preparation and Characterization of PH-Sensitive Hydrogels Based on Chitosan, Itaconic Acid and Methacrylic Acid. Polym. Int. 2011, 60 (3), 443–452. 10.1002/pi.2967.

[ref23] Sugama T.; Cook M. Poly(Itaconic Acid)-Modified Chitosan Coatings for Mitigating Corrosion of Aluminum Substrates. Prog. Org. Coat. 2000, 38 (2), 79–87. 10.1016/S0300-9440(00)00077-1.

[ref24] Jiang M.; Wang K.; Kennedy J. F.; Nie J.; Yu Q.; Ma G. Preparation and Characterization of Water-Soluble Chitosan Derivative by Michael Addition Reaction. Int. J. Biol. Macromol. 2010, 47 (5), 696–699. 10.1016/j.ijbiomac.2010.09.002. 20837054

[ref25] Silva S. M. L.; Braga C. R. C.; Fook M. V. L.; Raposo C. M. O.; Carvalho L. H.; Canedo E. L. Application of Infrared Spectroscopy to Analysis of Chitosan/Clay Nanocomposites. In Infrared Spectroscopy - Materials Science, Engineering and Technology; Theophile T., Ed.; InTech: 2012; pp 43–62. 10.5772/35522.

[ref26] Abou_Taleb M. H. Thermal and Spectroscopic Studies of Poly(N-Vinyl Pyrrolidone)/Poly(Vinyl Alcohol) Blend Films. J. Appl. Polym. Sci. 2009, 114 (2), 1202–1207. 10.1002/app.30082.

[ref27] Rinaudc M.; Pavlov G.; Desbrières J. Solubilization of Chitosan in Strong Acid Medium. Int. J. Polym. Anal. Charact. 1999, 5 (3), 267–276. 10.1080/10236669908009742.

[ref28] Domard A. PH and c.d. Measurements on a Fully Deacetylated Chitosan: Application to CuII—Polymer Interactions. Int. J. Biol. Macromol. 1987, 9 (2), 98–104. 10.1016/0141-8130(87)90033-X.

[ref29] Ramanery F. P.; Mansur A. A.; Mansur H. S. One-Step Colloidal Synthesis of Biocompatible Water-Soluble ZnS Quantum Dot/Chitosan Nanoconjugates. Nanoscale Res. Lett. 2013, 8 (1), 512 10.1186/1556-276X-8-512. 24308633 PMC4234014

[ref30] Maciel V. B. V.; Yoshida C. M. P.; Pereira S. M. S. S.; Goycoolea F. M.; Franco T. T. Electrostatic Self-Assembled Chitosan-Pectin Nano- and Microparticles for Insulin Delivery. Molecules 2017, 22 (10), 1707 10.3390/molecules22101707. PMC6151702 29023400

[ref31] Swain S. K.; Dey R. K.; Islam M.; Patel R. K.; Jha U.; Patnaik T.; Airoldi C. Removal of Fluoride from Aqueous Solution Using Aluminum-Impregnated Chitosan Biopolymer. Sep. Sci. Technol. 2009, 44 (9), 2096–2116. 10.1080/01496390902881212.

[ref32] Upadhyaya L.; Singh J.; Agarwal V.; Tewari R. P. Biomedical Applications of Carboxymethyl Chitosans. Carbohydr. Polym. 2013, 91 (1), 452–466. 10.1016/j.carbpol.2012.07.076. 23044156

[ref33] Bordenave N.; Grelier S.; Coma V. Advances on Selective C-6 Oxidation of Chitosan by TEMPO. Biomacromolecules 2008, 9 (9), 2377–2382. 10.1021/bm800375v. 18700797

[ref34] Li A.; Xue Q.; Ye Y.; Gong P.; Deng M.; Jiang B. Study on TEMPO-Mediated Oxidation of N-Succinyl Chitosan and the Water Retention Property. Molecules 2020, 25 (20), 4698 10.3390/molecules25204698. PMC7587373 33066471

[ref35] Zhang C.; Ping Q.; Zhang H.; Shen J. Synthesis and Characterization of Water-Soluble O-Succinyl-Chitosan. Eur. Polym. J. 2003, 39 (8), 1629–1634. 10.1016/S0014-3057(03)00068-5.

[ref36] Cabral J. D.; Roxburgh M.; Shi Z.; Liu L.; McConnell M.; Williams G.; Evans N.; Hanton L. R.; Simpson J.; Moratti S. C.; Robinson B. H.; Wormald P. J.; Robinson S. Synthesis, Physiochemical Characterization, and Biocompatibility of a Chitosan/Dextran-Based Hydrogel for Postsurgical Adhesion Prevention. J. Mater. Sci.: Mater. Med. 2014, 25 (12), 2743–2756. 10.1007/s10856-014-5292-3. 25085242

[ref37] Doshi B.; Hietala S.; Sirviö J. A.; Repo E.; Sillanpää M. A Powdered Orange Peel Combined Carboxymethyl Chitosan and Its Acylated Derivative for the Emulsification of Marine Diesel and 2T-Oil with Different Qualities of Water. J. Mol. Liq. 2019, 291, 111327 10.1016/j.molliq.2019.111327.

[ref38] Kalliola S.; Repo E.; Srivastava V.; Zhao F.; Heiskanen J. P.; Sirviö J. A.; Liimatainen H.; Sillanpää M. Carboxymethyl Chitosan and Its Hydrophobically Modified Derivative as PH-Switchable Emulsifiers. Langmuir 2018, 34 (8), 2800–2806. 10.1021/acs.langmuir.7b03959. 29406746 PMC6150725

[ref39] Cho E. J.; Doh K.-O.; Park J.; Hyun H.; Wilson E. M.; Snyder P. W.; Tsifansky M. D.; Yeo Y. Zwitterionic Chitosan for the Systemic Treatment of Sepsis. Sci. Rep. 2016, 6 (1), 29739 10.1038/srep29739. 27412050 PMC4944199

[ref40] Hyun H.; Hashimoto-Hill S.; Kim M.; Tsifansky M. D.; Kim C. H.; Yeo Y. Succinylated Chitosan Derivative Has Local Protective Effects on Intestinal Inflammation. ACS Biomater. Sci. Eng. 2017, 3 (8), 1853–1860. 10.1021/acsbiomaterials.7b00262. 29450257 PMC5810976

[ref41] Neagu V.; Vasiliu S.; Racovita S. Adsorption Studies of Some Inorganic and Organic Salts on New Zwitterionic Ion Exchangers with Carboxybetaine Moieties. Chem. Eng. J. 2010, 162 (3), 965–973. 10.1016/j.cej.2010.07.002.

[ref42] Xiang J.; Zhu R.; Lang S.; Yan H.; Liu G.; Peng B. Mussel-Inspired Immobilization of Zwitterionic Silver Nanoparticles toward Antibacterial Cotton Gauze for Promoting Wound Healing. Chem. Eng. J. 2021, 409, 128291 10.1016/j.cej.2020.128291.

[ref43] Deka M.; Saikia C. N. Chemical Modification of Wood with Thermosetting Resin: Effect on Dimensional Stability and Strength Property. Bioresour. Technol. 2000, 73 (2), 179–181. 10.1016/S0960-8524(99)00167-4.

[ref44] Sethi J.; Oksman K.; Illikainen M.; Sirviö J. A. Sonication-Assisted Surface Modification Method to Expedite the Water Removal from Cellulose Nanofibers for Use in Nanopapers and Paper Making. Carbohydr. Polym. 2018, 197, 92–99. 10.1016/j.carbpol.2018.05.072. 30007663

[ref45] Cazón P.; Vázquez M. Mechanical and Barrier Properties of Chitosan Combined with Other Components as Food Packaging Film. Environ. Chem. Lett. 2020, 18 (2), 257–267. 10.1007/s10311-019-00936-3.

[ref46] Brown N.; Ward I. M. Load Drop at the Upper Yield Point of a Polymer. J. Polym. Sci. Part A-2 Polym. Phys. 1968, 6 (3), 607–620. 10.1002/pol.1968.160060314.

[ref47] Hoy R. S.; Robbins M. O. Strain Hardening of Polymer Glasses: Effect of Entanglement Density, Temperature, and Rate. J. Polym. Sci., Part B: Polym. Phys. 2006, 44 (24), 3487–3500. 10.1002/polb.21012.

[ref48] Miwa Y.; Kurachi J.; Kohbara Y.; Kutsumizu S. Dynamic Ionic Crosslinks Enable High Strength and Ultrastretchability in a Single Elastomer. Commun. Chem. 2018, 1 (1), 1–8. 10.1038/s42004-017-0004-9.

[ref49] Henriksson M.; Berglund L. A.; Isaksson P.; Lindström T.; Nishino T. Cellulose Nanopaper Structures of High Toughness. Biomacromolecules 2008, 9 (6), 1579–1585. 10.1021/bm800038n. 18498189

[ref50] Sirviö J. A.; Ismail M. Y.; Zhang K.; Tejesvi M. V.; Ämmälä A. Transparent Lignin-Containing Wood Nanofiber Films with UV-Blocking, Oxygen Barrier, and Anti-Microbial Properties. J. Mater. Chem. A 2020, 8 (16), 7935–7946. 10.1039/C9TA13182E.

[ref51] Yang X.; Reid M. S.; Olsén P.; Berglund L. A. Eco-Friendly Cellulose Nanofibrils Designed by Nature: Effects from Preserving Native State. ACS Nano 2020, 14 (1), 724–735. 10.1021/acsnano.9b07659. 31886646

[ref52] Gao L.; Chao L.; Hou M.; Liang J.; Chen Y.; Yu H.-D.; Huang W. Flexible, Transparent Nanocellulose Paper-Based Perovskite Solar Cells. Npj Flex. Electron. 2019, 3 (1), 1–8. 10.1038/s41528-019-0048-2.

[ref53] Wang X.; Yao C.; Wang F.; Li Z. Cellulose-Based Nanomaterials for Energy Applications. Small 2017, 13 (42), 1702240 10.1002/smll.201702240. PMC5837049 28902985

[ref54] Tao J.; Wang R.; Yu H.; Chen L.; Fang D.; Tian Y.; Xie J.; Jia D.; Liu H.; Wang J.; Tang F.; Song L.; Li H. Highly Transparent, Highly Thermally Stable Nanocellulose/Polymer Hybrid Substrates for Flexible OLED Devices. ACS Appl. Mater. Interfaces 2020, 12 (8), 9701–9709. 10.1021/acsami.0c01048. 32013388

[ref55] Sirviö J. A.; Kolehmainen A.; Visanko M.; Liimatainen H.; Niinimäki J.; Hormi O. E. O. Strong, Self-Standing Oxygen Barrier Films from Nanocelluloses Modified with Regioselective Oxidative Treatments. ACS Appl. Mater. Interfaces 2014, 6 (16), 14384–14390. 10.1021/am503659j. 25089516

[ref56] Sethi J.; Farooq M.; Sain S.; Sain M.; Sirviö J. A.; Illikainen M.; Oksman K. Water Resistant Nanopapers Prepared by Lactic Acid Modified Cellulose Nanofibers. Cellulose 2018, 25 (1), 259–268. 10.1007/s10570-017-1540-2.

[ref57] Sharma A.; Thakur M.; Bhattacharya M.; Mandal T.; Goswami S. Commercial Application of Cellulose Nano-Composites – A Review. Biotechnol. Rep. 2019, 21, e00316 10.1016/j.btre.2019.e00316. PMC6389799 30847286

[ref58] Tang R.; Yu Z.; Renneckar S.; Zhang Y. Coupling Chitosan and TEMPO-Oxidized Nanofibrilliated Cellulose by Electrostatic Attraction and Chemical Reaction. Carbohydr. Polym. 2018, 202, 84–90. 10.1016/j.carbpol.2018.08.097. 30287046

[ref59] Yang W.; Bian H.; Jiao L.; Wu W.; Deng Y.; Dai H. High Wet-Strength, Thermally Stable and Transparent TEMPO-Oxidized Cellulose Nanofibril Film via Cross-Linking with Poly-Amide Epichlorohydrin Resin. RSC Adv. 2017, 7 (50), 31567–31573. 10.1039/C7RA05009G.

[ref60] Wang J.; Gardner D. J.; Stark N. M.; Bousfield D. W.; Tajvidi M.; Cai Z. Moisture and Oxygen Barrier Properties of Cellulose Nanomaterial-Based Films. ACS Sustainable Chem. Eng. 2018, 6 (1), 49–70. 10.1021/acssuschemeng.7b03523.

[ref61] Bastarrachea L.; Dhawan S.; Sablani S. S. Engineering Properties of Polymeric-Based Antimicrobial Films for Food Packaging: A Review. Food Eng. Rev. 2011, 3 (2), 79–93. 10.1007/s12393-011-9034-8.

[ref62] Sirviö J. A.; Honkaniemi S.; Visanko M.; Liimatainen H. Composite Films of Polyvinyl Alcohol and Bifunctional Crosslinking Cellulose Nanocrystals. ACS Appl. Mater. Interfaces 2015, 7 (35), 19691–19699. 10.1021/acsami.5b04879. 26280660

[ref63] Rindlav-Westling A.; Stading M.; Hermansson A.-M.; Gatenholm P. Structure, Mechanical and Barrier Properties of Amylose and Amylopectin Films. Carbohydr. Polym. 1998, 36 (2), 217–224. 10.1016/S0144-8617(98)00025-3.

[ref64] Shimizu M.; Saito T.; Isogai A. Water-Resistant and High Oxygen-Barrier Nanocellulose Films with Interfibrillar Cross-Linkages Formed through Multivalent Metal Ions. J. Membr. Sci. 2016, 500, 1–7. 10.1016/j.memsci.2015.11.002.

[ref65] Syverud K.; Stenius P. Strength and Barrier Properties of MFC Films. Cellulose 2009, 16 (1), 75 10.1007/s10570-008-9244-2.

[ref66] Visanko M.; Liimatainen H.; Sirviö J. A.; Mikkonen K. S.; Tenkanen M.; Sliz R.; Hormi O.; Niinimäki J. Butylamino-Functionalized Cellulose Nanocrystal Films: Barrier Properties and Mechanical Strength. RSC Adv. 2015, 5 (20), 15140–15146. 10.1039/C4RA15445B.

[ref67] Sirviö J. A.; Hyypiö K.; Asaadi S.; Junka K.; Liimatainen H. High-Strength Cellulose Nanofibers Produced via Swelling Pretreatment Based on a Choline Chloride–Imidazole Deep Eutectic Solvent. Green Chem. 2020, 22 (5), 1763–1775. 10.1039/C9GC04119B.

[ref68] Aulin C.; Gällstedt M.; Lindström T. Oxygen and Oil Barrier Properties of Microfibrillated Cellulose Films and Coatings. Cellulose 2010, 17 (3), 559–574. 10.1007/s10570-009-9393-y.

[ref69] Kumar V.; Bollström R.; Yang A.; Chen Q.; Chen G.; Salminen P.; Bousfield D.; Toivakka M. Comparison of Nano- and Microfibrillated Cellulose Films. Cellulose 2014, 21 (5), 3443–3456. 10.1007/s10570-014-0357-5.

[ref70] Corazzari I.; Nisticò R.; Turci F.; Faga M. G.; Franzoso F.; Tabasso S.; Magnacca G. Advanced Physico-Chemical Characterization of Chitosan by Means of TGA Coupled on-Line with FTIR and GCMS: Thermal Degradation and Water Adsorption Capacity. Polym. Degrad. Stab. 2015, 112, 1–9. 10.1016/j.polymdegradstab.2014.12.006.

[ref71] C.-Eulalio H. Y.; B.-Rodrigues J. F.; O.-Santos K.; Peniche C.; V.-LiaFook M. Characterization and Thermal Properties of Chitosan Films Prepared with Different Acid Solvents. Rev. Cuba. Quím. 2019, 31 (3), 309–323.

[ref72] Ziegler-Borowska M.; Chełminiak D.; Kaczmarek H.; Kaczmarek-Kędziera A. Effect of Side Substituents on Thermal Stability of the Modified Chitosan and Its Nanocomposites with Magnetite. J. Therm. Anal. Calorim. 2016, 124 (3), 1267–1280. 10.1007/s10973-016-5260-x.

[ref73] de Britto D.; Campana-Filho S. P. A Kinetic Study on the Thermal Degradation of N,N,N-Trimethylchitosan. Polym. Degrad. Stab. 2004, 84 (2), 353–361. 10.1016/j.polymdegradstab.2004.02.005.

[ref74] Botelho da Silva S.; Krolicka M.; van den Broek L. A. M.; Frissen A. E.; Boeriu C. G. Water-Soluble Chitosan Derivatives and PH-Responsive Hydrogels by Selective C-6 Oxidation Mediated by TEMPO-Laccase Redox System. Carbohydr. Polym. 2018, 186, 299–309. 10.1016/j.carbpol.2018.01.050. 29455991

[ref75] Bukzem A. L.; Signini R.; dos Santos D. M.; Lião L. M.; Ascheri D. P. R. Optimization of Carboxymethyl Chitosan Synthesis Using Response Surface Methodology and Desirability Function. Int. J. Biol. Macromol. 2016, 85, 615–624. 10.1016/j.ijbiomac.2016.01.017. 26778157

[ref76] Miranda M. E. S.; Marcolla C.; Rodrígues C. A.; Wilhelm H. M.; Sierakowski M. R.; Bresolin T. M. B.; de Freitas R. A. Chitosan and N-Carboxymethylchitosan: I. The Role of N-Carboxymethylation of Chitosan in the Thermal Stability and Dynamic Mechanical Properties of Its Films. Polym. Int. 2006, 55 (8), 961–969. 10.1002/pi.2060.

[ref77] Wu X.; Yang C. Q.; He Q. Flame Retardant Finishing of Cotton Fleece: Part VII. Polycarboxylic Acids with Different Numbers of Functional Group. Cellulose 2010, 17 (4), 859–870. 10.1007/s10570-010-9416-8.

[ref78] Ren Y.; Tian T.; Jiang L.; Guo Y. Fabrication of Chitosan-Based Intumescent Flame Retardant Coating for Improving Flame Retardancy of Polyacrylonitrile Fabric. Molecules 2019, 24 (20), 3749 10.3390/molecules24203749. PMC6833438 31627459

