# Supplementary material for: Aqueous Modification of Chitosan with Itaconic Acid to Produce Strong Oxygen Barrier Film
Source: Biomacromolecules. 2021 Apr 29;22(5):2119–28. doi: 10.1021/acs.biomac.1c00216 (PMC8382240; doi:10.1021/acs.biomac.1c00216)
Supplement: Supplementary file 1 — bm1c00216_si_001.pdf [file bm1c00216_si_001.pdf]

Aqueous modification of chitosan with itaconic acid to produce strong oxygen barrier film

Juho Antti Sirviö,[a]* Anu Kantola[b], Sanna Komulainen[b], Svitlana Filonenko[c]

[a] Fibre and Particle Engineering Research Unit, University of Oulu, P.O. Box 4300, 90014 Oulu, Finland E-mail: juho.sirvio@oulu.fi.
[b] NMR Research Unit, University of Oulu, P.O.Box 3000, FIN-90014, Oulu, Finland.
[c] Max Planck Institute of Colloids and Interfaces, Research Campus Golm, 14424 Potsdam, Germany

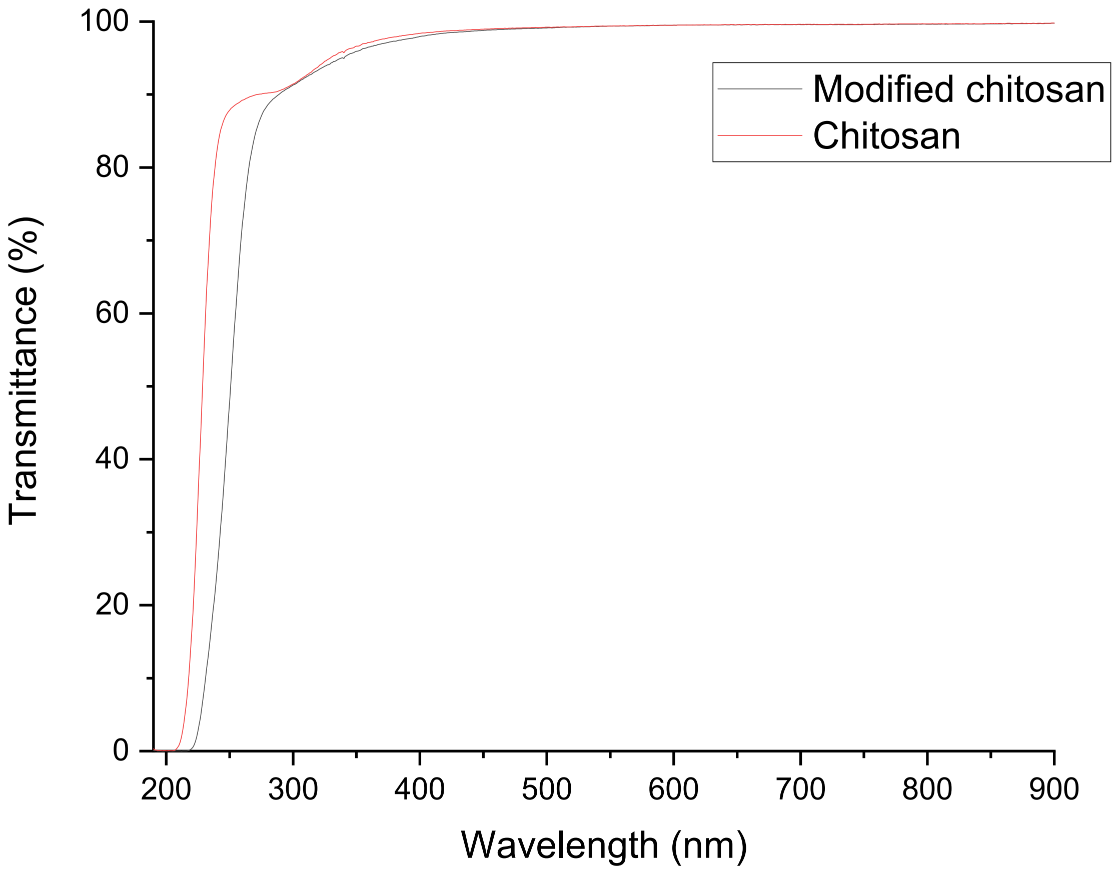

Figure S1. UV Vis spectra of original and modified chitosan solutions in water (0.1%)

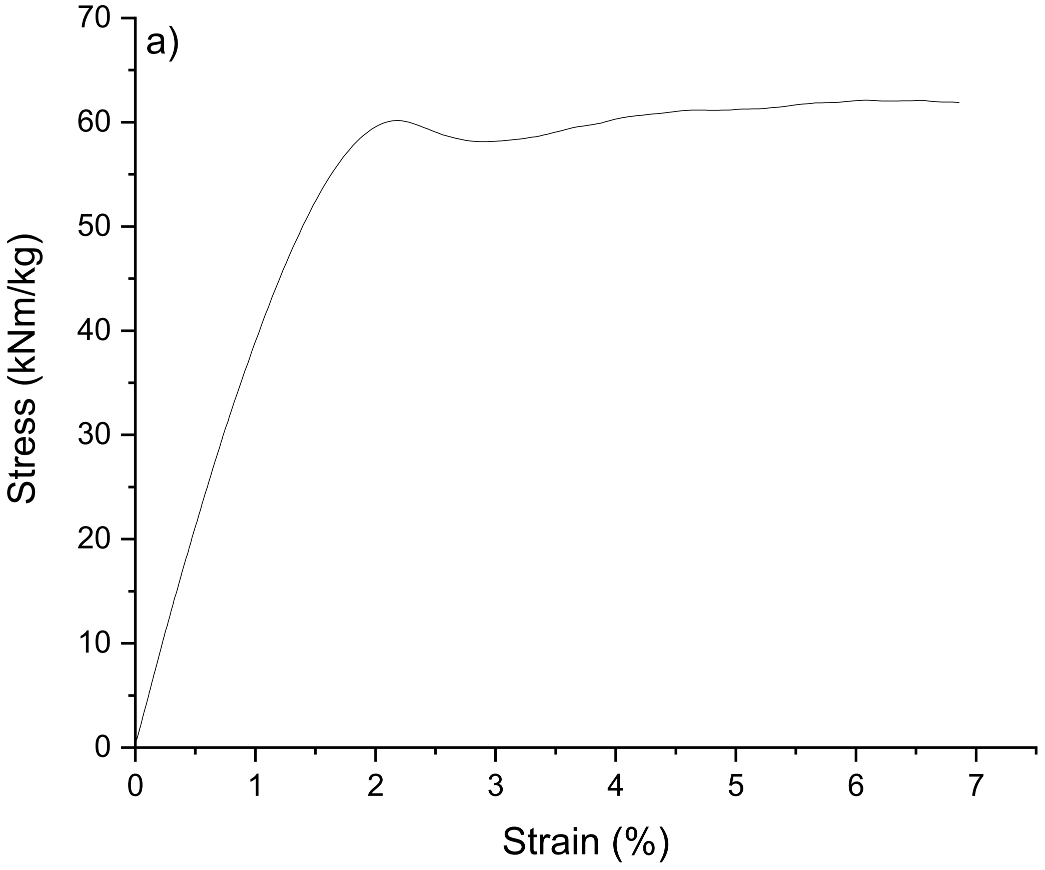
a)
Stress (kNm/kg)
Strain (%)
0
10
20
30
40
50
60
70
0
1
2
3
4
5
6
7

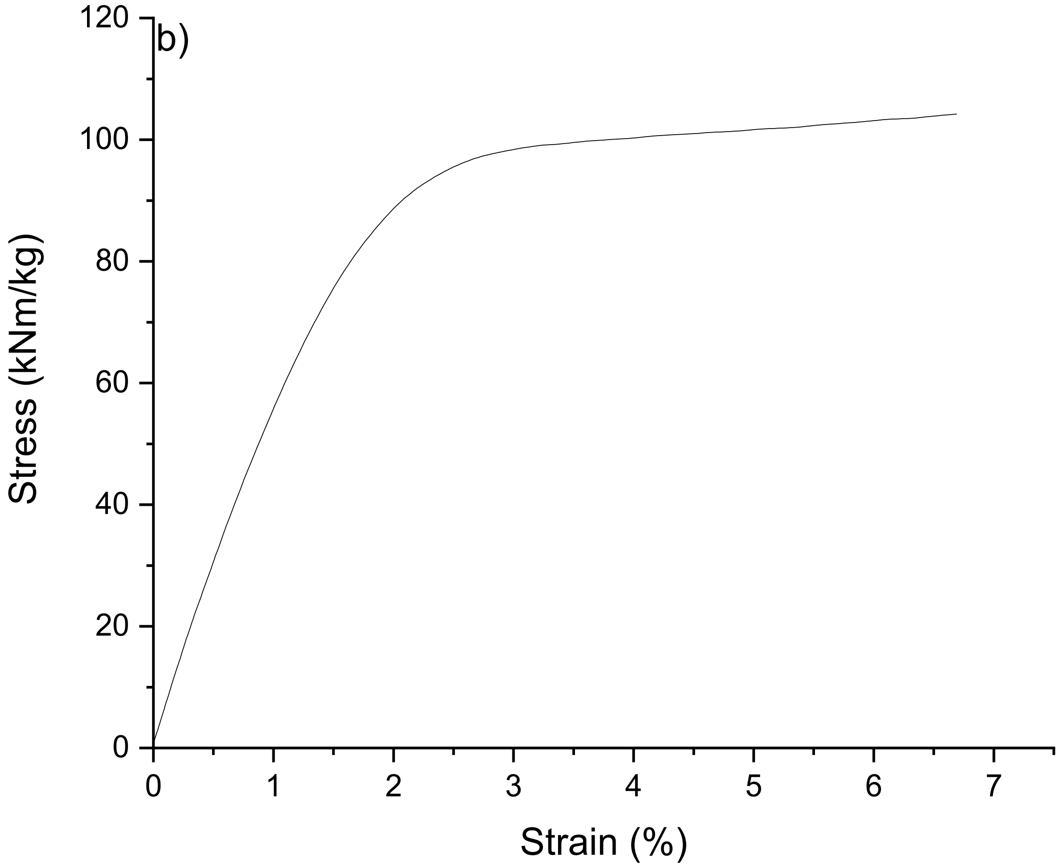

Figure S2. Examples of stress-strain curves of a) original and b) modified chitosan films.
